# Identification of Major Depressive Disorder Using Multiple Functional Connection Patterns

**DOI:** 10.1002/cns.70951

**Published:** 2026-05-28

**Authors:** Yudi Ruan, Lihe Guan, Liling Peng, Leina Zhao, Yufen Huang, Weikai Li, Xin Gao

**Affiliations:** ^1^ College of Mathematics and Statistics Chongqing Jiaotong University Chongqing China; ^2^ Key Laboratory of Complex Systems Optimization and Intelligent Control of Chongqing Municipal Education Commission Chongqing Jiaotong University Chongqing China; ^3^ Department of PET/MR Shanghai Universal Medical Imaging Diagnostic Center Shanghai China; ^4^ Digital Medical Research Institute, School of Medicine Shanghai University Shanghai China; ^5^ Department of Radiology Shaoxing Maternity and Child Health Care Hospital Shaoxing China

**Keywords:** connection patterns, functional connectivity network, graph convolutional network, major depressive disorder, resting‐state fMRI

## Abstract

**Background:**

Major depressive disorder (MDD) poses a significant challenge to global mental health, highlighting the urgent need for effective diagnostic tools to enable early and accurate detection. Resting‐state functional magnetic resonance imaging (rs‐fMRI) has garnered considerable attention for evaluating MDD through functional connectivity networks (FCNs). While graph convolutional networks (GCNs) have advanced FCN analysis by capturing complex interregional connection patterns, existing GCN‐based approaches have predominantly focused on a single connection pattern, thereby neglecting the multifaceted information encoded in diverse connection profiles.

**Methods:**

We propose a Multiple Functional Connection Patterns Graph Convolutional Network (MFCP) that integrates three distinct connection patterns—sparse representation, Pearson correlation, and Granger causality mapping—to leverage their complementary information. The proposed framework employs multiple graph convolutional modules to integrate diverse connectivity information, thereby enhancing MDD‐related diagnostic features extracted from FCNs. We conducted primary experiments on the REST‐MDD dataset Site 20, comprising 533 subjects, to evaluate the proposed MFCP.

**Results:**

The MFCP framework achieved superior diagnostic performance with an accuracy of 87.74%, precision of 86.21%, recall of 90.91%, F1‐score of 88.50%, and an area under the ROC curve (AUC) of 0.9326. Comparative analysis revealed that integrating three connection patterns significantly outperformed single‐pattern approaches (accuracy range: 72.64%–81.13%) and two‐pattern combinations (accuracy range: 77.36%–83.02%). t‐SNE visualization confirmed enhanced class separability with increasing pattern integration, while Grad‐CAM analysis identified distinct discriminative brain regions across different connectivity patterns.

**Conclusions:**

The proposed MFCP framework effectively integrates multiple functional connection patterns to enhance diagnostic feature extraction, demonstrating strong effectiveness for MDD identification. These findings suggest that leveraging complementary connectivity information through multi‐pattern integration represents a promising approach for improving automated MDD diagnosis based on rs‐fMRI functional connectivity analysis.

## Graphical abstract

We propose a Multiple Functional Connection Pattern Graph Convolutional Network (MFCP) for major depressive disorder identification using resting‐state fMRI. By integrating Pearson correlation, sparse representation, and Granger causality–based brain networks, MFCP captures complementary connectivity information. Experiments on the REST‐MDD dataset demonstrate improved diagnostic performance with an accuracy of 87.74% and an AUC of 0.9326, while Grad‐CAM visualization highlights discriminative brain regions associated with MDD.

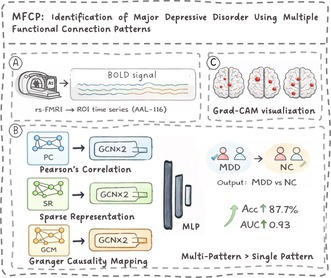

## 1 Introduction

Major Depressive Disorder is a severe illness characterized by persistent feelings of sadness, lack of interest, difficulties with thinking, and physical symptoms such as changes in sleep or appetite [1, 2]. MDD is the primary cause of disability and illness globally, impacting around 10% of the world's population [3]. According to data from the World Health Organization, nearly 10% to 15% of people will experience clinical depression at some point in their lives [4], with approximately 5% of men and 9% of women experiencing depression each year [5]. This lack of precise diagnostic tools makes it challenging for clinicians to accurately identify and treat these mood disorders at an early stage. Consequently, there is an urgent need to explore and develop translational biomarkers that can aid in the early diagnosis and differentiation of mood disorders. Leveraging advanced machine learning techniques holds great promise in identifying such biomarkers, thereby potentially transforming the diagnostic process and improving patient outcomes.

Currently, the convergence of deep learning methodologies with functional magnetic resonance imaging (fMRI) has emerged as a powerful tool for the diagnosis of MDD, advancing precision medicine in mental health [6]. Deep learning has the potential to reveal hidden information within fMRI data, leading to improved diagnosis and treatment of neurological diseases [7]. However, relying solely on fMRI data may not be sufficient due to the variability in brain activity and temporal differences among subjects. Resting‐state fMRI (rs‐fMRI), which does not require specific tasks from subjects, can eliminate task‐related variables and provide a functional connection map of the brain at rest, thus enhancing the accuracy of MDD diagnosis [8]. This is particularly important for MDD patients who may experience attention deficits and fatigue during task performance, potentially affecting the accuracy of fMRI data.

Utilizing rs‐fMRI to map functional connectivity networks has provided new horizons in the classification of brain disorders. This approach effectively identifies alterations in network configurations and the nuances of connectivity, distinguishing healthy connectivity patterns from pathological alterations, with each region of interest (ROI) serving as a pivotal node and the strength of connections as critical edges [9, 10]. The study of spontaneous activity fluctuations across different brain regions can provide insight into FCNs, helping to understand the underlying pathological mechanisms of MDD [11]. However, it is challenging to build FCNs for predicting MDD, as it requires extracting network features [12, 13] and heavily relies on domain expertise [2]. Graph Convolutional Networks (GCNs) are a highly effective deep learning method that captures both local and global information from neighboring nodes in a hierarchical manner, allowing for the automatic learning of graph representations in a data‐centric approach [14, 15, 16, 17]. The advancement of GCNs has proven to be an efficient technique for identifying fMRI biomarkers within FCNs and expediting the diagnostic process [18]. Essentially, every brain network mirrors a sophisticated graph, a complex graph consisting of individual nodes and their interwoven topological relationships [19]. GCNs, as a cutting‐edge deep learning architecture, excel at automatically extracting intrinsic node features and decoding the complex topological connections between them [20]. Through the application of advanced techniques such as convolution and graph pooling, GCNs are capable of analyzing fMRI data and extracting advanced topological data from brain networks, thereby leading to a significant improvement in the accuracy of diagnosing brain diseases [21]. To dissect FCNs and map the intricate workings of the human brain, numerous studies have delineated the cerebral cortex into discrete ROIs. Employing GCN models, they have effectively extracted meaningful features from these ROIs, thereby enhancing the diagnosis within the realm of FCNs [22, 23]. Previous research has predominantly focused on the synchronous topological traits and connection weights within FCNs, derived from single connection patterns. Unfortunately, this method falls short of revealing the full spectrum of complementary insights that diverse connection patterns within FCNs can offer.

**FIGURE 1:**
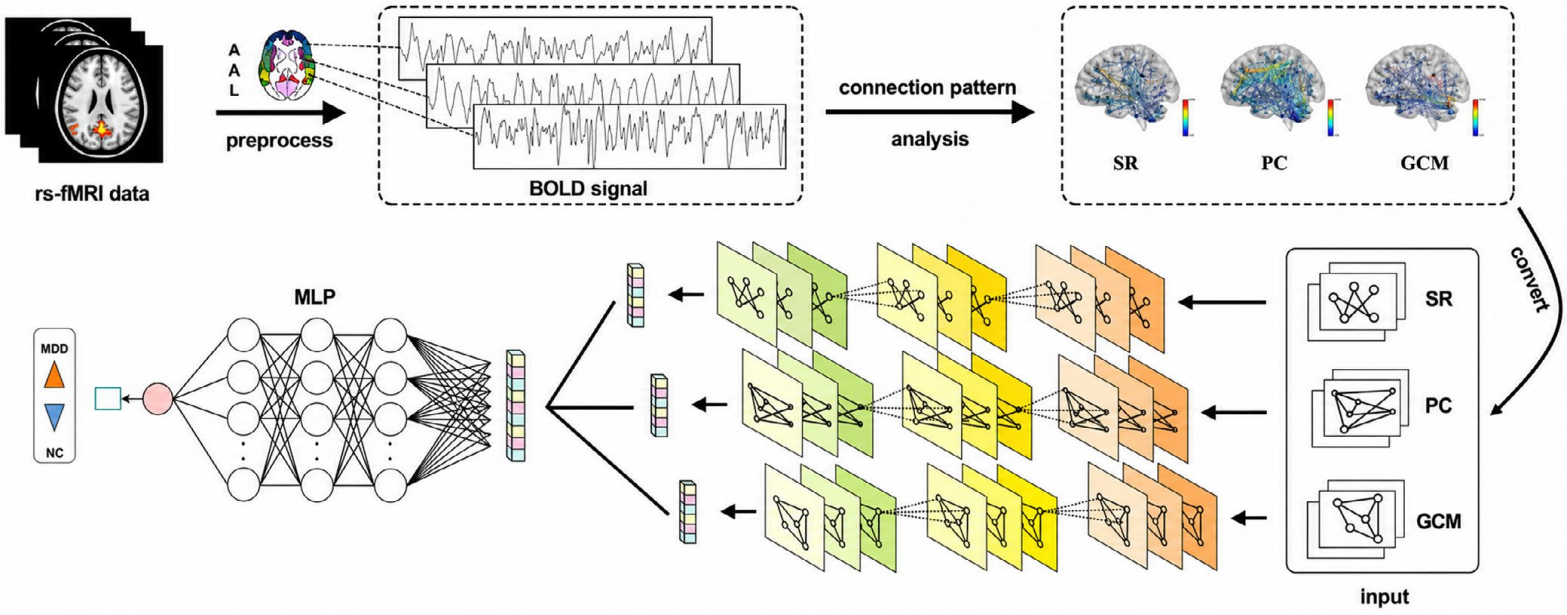
The complete process of the Multiple Functional Connection Patterns Network for diagnosing MDD.

To address this limitation, we introduce the Multiple Functional Connection Patterns Graph Convolutional Network (MFCP), a novel diagnostic approach for MDD utilizing rs‐fMRI. As depicted in Figure 1, our method begins by constructing multiple connectivity graphs per subject, each encapsulating a unique FCN. The brain is segmented into 116 distinct ROIs via the Automated Anatomical Labeling (AAL) atlas, followed by the construction of brain networks through three complementary techniques: Pearson's Correlation (PC), Sparse Representation (SR), and Granger Causality Mapping (GCM). Specifically, PC captures synchronous inter‐regional co‐fluctuations, SR emphasizes sparse conditional dependencies to reduce indirect or noisy connections, and GCM characterizes directed effective interactions with temporal precedence; these patterns thus provide complementary information about brain connectivity. Subsequently, we employ parallel GCN modules to learn graph representations from each pattern, as GCNs are well‐suited to exploit the intrinsic graph structure of FCNs by aggregating topological neighborhood information. The resulting multi‐pattern features are subsequently refined through a series of three fully connected layers, yielding a diagnosis based on the final layer's output. To validate the MFCP approach's efficacy, we examined rs‐fMRI scans from 533 individuals within the REST‐MDD dataset. Our comparative analysis revealed that integrating various connection patterns significantly outperformed single‐pattern methods, markedly enhancing the precision of MDD diagnosis with our MFCP methodology.

## 2 Related Works

### 2.1 Graph Convolutional Networks

GCNs are gaining prominence in disease diagnosis and classification, owing to their proficiency at processing graph‐structured data. In a pioneering study, Parisot et al. leveraged a GCN to model the brain as a sparse graph, merging imaging‐derived feature vectors with phenotypic data as edge weights, thereby advancing the diagnosis of neurological diseases [24]. Pan et al. introduced a multi‐scale adaptive multi‐channel fusion deep graph convolutional network with an attention mechanism (MAMF‐GCN) to effectively predict mental disorders by better integrating features of modalities and different atlases by exploiting multi‐channel correlations [25]. Sun et al. presented a new graph convolutional network based on complex networks to identify MDD using multichannel EEG signals [26]. The combination of GCNs with multi‐view techniques has significantly improved classification performance. Zhao et al. proposed a novel method for enhancing multi‐view brain network features based on self‐attention mechanism graph convolutional networks (SA‐GCN), which enhances node features through connections between different nodes and extracts deeper and more discriminative features [27]. Song et al. introduced a module for multi‐view attention fusion to extract useful information from different views, advancing the diagnostic capabilities of GCNs for Autism Spectrum Disorder (ASD) [28]. Rather than relying on a single view, employing multi‐view approaches significantly improves the classification accuracy of models.

### 2.2 Multi‐View Learning

Multi‐view learning, a sophisticated machine learning paradigm, capitalizes on the inherent diversity of real‐world data. It effectively handles instances characterized by multiple feature sets, which naturally enhances our ability to extract richer insights and enable more detailed analyses. Additionally, multi‐view techniques are widely used in the medical field. Influenced by the concept of multi‐view learning, which involves utilizing information from various perspectives to improve feature representation, Huang et al. [6], developed a new framework that enhances the representation of FCNs by combining both common and complementary information from multiple networks [29]. Wang et al. have recently introduced the integration of PET/MRI with fMRI in the surgical assessment of mTLE‐HS patients, targeting the prediction of adverse outcomes. Their innovative approach has delivered promising predictive insights [12]. Compared to single‐view methods, multi‐view approaches offer the advantage of incorporating diverse perspectives and information sources, leading to a more comprehensive understanding of the data. By leveraging multiple views, these approaches can capture a richer representation of the underlying patterns and relationships within the dataset. By integrating complementary information from different views, multi‐view models often achieve better classification performance than single‐view approaches. Additionally, multi‐view methods have been shown to be more robust in handling complex and high‐dimensional data, making them particularly well‐suited for neuroimaging analysis. Overall, the use of multi‐view approaches represents a promising direction for improving model performance across various domains.

## 3 Materials and Methods

### 3.1 Data Acquisition

In this study, we utilized the publicly accessible Resting‐State fMRI Data Analysis for Major Depressive Disorder (REST‐MDD) dataset [30], specifically sites 20 and 21. This multi‐site dataset is a valuable resource for investigating MDD, as it includes rs‐fMRI data from both MDD patients and normal controls across independent data collection centers. Specifically, site 20 comprises rs‐fMRI scans from 282 individuals diagnosed with MDD and 251 normal controls, while site 21 includes 86 MDD patients and 70 NCs. Primary analyses were conducted using site 20 due to its larger sample size, which enhances the statistical power of our findings. In addition, site 21 was employed as an independent validation set to assess the cross‐site generalizability of the proposed framework. The demographic information of the subjects is given in Table 1. Age and gender distributions were compared between the MDD and NC groups using a Welch's *t*‐test and a Pearson chi‐square test, respectively, and no significant between‐group differences were observed. Based on this well‐balanced dataset, we further evaluated the effectiveness of the proposed MFCP framework for MDD diagnosis.

**TABLE 1:** Demographic information of the subjects.

Site	Characteristic	MDD	NC	*p*
20	$\text{Gender}\left(M/F\right)$	99/183	87/164	0.987
	$\mathrm{Age}\left(\text{year}\pm \mathrm{SD}\right)$	38.74±13.74	39.6±15.87	0.487
21	$\text{Gender}\left(M/F\right)$	38/48	31/39	0.999
	$\mathrm{Age}\left(\text{year}\pm \mathrm{SD}\right)$	34.71 ± 12.56	36.13 ± 12.55	0.483

### 3.2 Data Preprocessing

The rs‐fMRI scans were conducted using a standard echo‐planar imaging sequence on a Siemens Tim Trio 3 T scanner, which is commonly utilized in clinical environments. The preprocessing pipeline detailed below was applied to the Site 20 dataset, with identical procedures subsequently performed on Site 21 data. Participants were directed to relax and fixate their gaze on a white fixation cross presented against a black background throughout the 6‐min scan. The imaging parameters were configured as follows: A flip angle of 90 degrees, acquisition of 32 slices, a repetition time (TR) of 2000 ms, an echo time (TE) of 30 ms, collection of 242 volumes, and a slice thickness of 3.0 mm. For data preprocessing, we utilized the widely accepted Statistical Parametric Mapping software (SPM8). To reduce signal artifacts, the first 10 volumes from each subject's rs‐fMRI scans were discarded. The remaining images underwent the subsequent preprocessing steps: (1) normalization to the MNI space with a resolution of $3\times 3\times 3mm^{3}$; (2) nuisance signal regression to control for signals originating from the ventricles, white matter, global signals, and head motion, utilizing Friston's 24‐parameter model [31]. (3) band‐pass filtering within the frequency range of 0.01–0.08 Hz; (4) extraction of BOLD time series from 116 ROIs defined by the AAL atlas. Subsequently, these time series were organized into a data matrix, referred to as $X\in R^{232\times 116}.$

### 3.3 Proposed Method

In this section, we introduce our proposed Multiple Functional Connection Patterns approach for the automatic diagnosis of MDD. Our approach consists of three main components, each critical to the overall framework: (1) Creating FCNs with Multiple Patterns: We utilize the AAL atlas, which includes 116 regions of interest, to construct FCNs. By leveraging multiple connection patterns, we capture diverse aspects of brain network interactions, providing a comprehensive representation of functional connectivity. (2) Producing graph representations for each individual based on multiple patterns: For each individual, we generate graph representations corresponding to the different connection patterns identified in the first step. These graphs encapsulate the intricate relationships between the ROIs, reflecting the unique functional connection characteristics of each individual's brain. (3) Identifying MDD through integration and classification of multi‐pattern features: We integrate the multi‐pattern features derived from the graph representations to create a unified feature set. This integrated feature set is then processed through a series of GCN modules, followed by fully connected layers, to perform classification. The final output is used to determine the presence of MDD, leveraging the combined information from all connection patterns to improve diagnostic accuracy.

#### 3.3.1 Multi‐Pattern Brain Network Construction

Consider an undirected graph $G=\left\{V,E,A\right\},$ where V denotes the set of nodes consisting of n nodes, E represents the set of edges, and A is an n×n adjacency matrix that represents the connectivity patterns among the nodes within a specific FCN. In this research, we build brain networks using the AAL atlas, employing three different connection patterns. These patterns are as follows:

**(1) Pearson Correlation**.

In a functional connection matrix, the connection between two ROIs is represented by the Pearson correlation coefficient derived from their respective average time series signals. The connection weight $w_{\mathrm{ij}}$ between the i− th and j‐th ROIs is defined as follows:

(1) $$w_{\mathrm{ij}}=\frac{{\left(x_{i}-{\bar{x}}_{i}\right)}^{T}\left(x_{j}-{\bar{x}}_{j}\right)}{\sqrt{{\left(x_{i}-{\bar{x}}_{i}\right)}^{T}\left(x_{i}-{\bar{x}}_{i}\right)}\sqrt{{\left(x_{j}-{\bar{x}}_{j}\right)}^{T}\left(x_{j}-{\bar{x}}_{j}\right)}},$$

here, we define $x_{i}\in R^{t}$ as the vector of a sequence of BOLD signals derived from the i− th ROI, where t represents the total number of time points. The term $\bar{x_{i}}\in R^{t}$ denotes the mean of $x_{i},$ where $\left(\;i=1,2,\ldots ,n\;\right).$ The expression $\left(x_{i}-\bar{x_{i}}\right)$ represents the centered form of $x_{i}$.

**(2) Sparse Representation**.

Unlike PC, which quantifies the overall correlation between two variables, Sparse Representation (SR) is a widely adopted method for modeling partial correlations between variables. This technique allows for the identification of direct dependencies within the data, capturing connections that may not be apparent through traditional correlation measures. As described by Li et al. [16], SR has become a crucial tool in various fields, particularly in scenarios where understanding the intricate dependencies between variables is essential. The SR model is expressed as follows:

(2) $$\min_{W}\sum_{i=1}^{n}{\left\|x_{i}-\sum_{j\neq i}W_{\mathrm{ij}}x_{j}\right\|}^{2}+\lambda \sum_{j\neq i}\left|W_{\mathrm{ij}}\right|,$$

where $X=\left[x_{1},x_{2},\cdots ,x_{n}\right]\in R^{t\times n}$ denotes the fMRI data matrix for a specific subject. Each column of X corresponds to the time course of an individual brain region.

**(3) Granger Causality Mapping**.

GCM is a sophisticated method used for modeling effective connection patterns within a network, specifically designed to uncover and analyze causal relationships between different nodes. Unlike traditional methods that typically assume symmetrical connections, GCM identifies directional, asymmetrical connections, thereby providing deeper insights into the cause‐and‐effect dynamics within the network. Specifically, given two time series $x\left[n\right]$ and $y\left[n\right]$ the GCM from $x\left[n\right]$ to $y\left[n\right]$ is defined as follows:

(3) $$F_{x,y}=\ln\frac{\left|\sum \left(\zeta_{t}\right)\right|}{\left|\sum \left(\eta_{t}\right)\right|},$$

where $\zeta_{t}$ and $\eta_{t}$ represent the residuals from the unrestricted and restricted regression models, respectively. The symbol Σ denotes the variance.

Since GCM produces a directed causality matrix, while the spectral GCN backbone in our framework takes a single non‐negative adjacency matrix, we convert the directed GCM into an undirected graph by aggregating bidirectional causality strengths. Specifically, for each ROI pair $\left(i,j\right)$, we define the GCM‐based adjacency weight as

(4) $$A_{\mathrm{ij}}^{\mathrm{GCM}}=|G_{i\to j}+G_{j\to i}|,i\neq j,$$

and set $A_{\mathrm{ii}}^{\mathrm{GCM}}=0$. This yields a symmetric and non‐negative adjacency matrix, which is then used to compute the normalized Laplacian for graph convolution.

To define the edges of the FCNs, we quantify the connectivity between each ROI and all other brain regions in the network. This methodology is based on the assumption that edge weights are inherently positive, as suggested by structural equilibrium theory as articulated by Cartwright [32] and Heider [33]. This assumption guarantees a balanced network structure with positive edge weights, aiding in the subsequent analysis of FCNs and convolution operations, as many brain connection indicators, such as mutual information [34] are non‐negative. The connection weight $e_{\mathrm{ij}}$ measures the strength of direct connections between brain regions, encompassing both positive and negative correlations. The n− dimensional vector $\left(\left|e_{i1}\right|,\left|e_{i2}\right|,\ldots ,\left|e_{\text{in}}\right|\right)$, where n=116, represents the level of connection between the i‐th brain region and other regions. Accordingly, we define the adjacency matrices $A_{\mathrm{PC}}^{\left(116\right)}\in R^{116\times 116},$ $A_{\mathrm{SR}}^{\left(116\right)}\in R^{116\times 116},$ and $A_{\mathrm{GCM}}^{\left(116\right)}\in R^{116\times 116}$ based on the three connection modes. We define $A\in \left\{A_{\mathrm{PC}}^{\left(116\right)},A_{\mathrm{SR}}^{\left(116\right)},A_{\mathrm{GCM}}^{\left(116\right)}\right\}$ Consequently, three connection patterns are used to construct three graphs, namely $G_{\mathrm{PC}}=G\left(X_{\mathrm{PC}},A_{\mathrm{PC}}\right),$ $G_{\mathrm{SR}}=G\left(X_{\mathrm{SR}},A_{\mathrm{SR}}\right)$, and $G_{\mathrm{GCM}}=G\left(X_{\mathrm{GCM}},A_{\mathrm{GCM}}\right)$, for each subject.

#### 3.3.2 Developing Graph Representations by Integrating Multiple Patterns

In this section, we conceptualize FCNs as non‐uniform graphs that possess intricate internal structures. To effectively analyze these FCNs and to develop innovative graph‐based representations for the diagnosis of MDD, we employ spectral GCNs. This approach is grounded in the methodology introduced by Zhang et al. [35], which leverages the power of spectral graph theory to capture the complex patterns and relationships within the FCNs. The spectral GCN utilizes the Fourier transform and its inverse to efficiently aggregate information among nodes within the spectral domain, following the techniques proposed by Bruna et al. [36] and Kawahara et al. [37].

The Laplacian matrix L is employed in the graph's Fourier transform and is defined as L=D−A, where D is the diagonal matrix with each diagonal element $D_{\mathrm{ii}}$ representing the degree of the i− th node. Specifically, $D_{\mathrm{ii}}$ is calculated by summing the weights of all edges connected to the i− th node, i.e., $D_{\mathrm{ii}}=\sum_{j}A_{\mathrm{ij}}$. A commonly used variant of the Laplacian matrix is the normalized symmetric Laplacian matrix:

(5) $$L=D^{-\frac{1}{2}}LD^{-\frac{1}{2}}=I_{n}-D^{-\frac{1}{2}}AD^{-\frac{1}{2}},$$

where $I_{n}$ is an identity matrix.

The decomposition of the Laplacian matrix L can be expressed as $U\Lambda U^{T}$, where $U={\left\{u_{i}\right\}}_{i=1}^{n}$ represents the orthogonal eigenvectors, $\Lambda =\mathrm{diag}{\left\{\alpha_{i}\right\}}_{i=1}^{n}$ is a diagonal matrix, and $\alpha_{i}$ denotes the eigenvalues associated with L. The matrix U facilitates the transformation of variables into the spectral domain, allowing convolutional operations on the graph [38].

The multiplication of the Fourier transforms of signals is equivalent to the multiplication of the Fourier transforms of the signals themselves, as demonstrated by Shuman et al. [39]. In the context of graph convolution, signals in the node domain are denoted as aandb. The definition of graph convolution can be formulated as follows:

(6) $$a*b=U\left(U^{T}b\right)\left(U^{T}a\right),$$

where * denotes the convolution operation.

ChebyNet [40] was introduced to reduce computational complexity by leveraging the graph convolution definition described earlier (Equation 6). To simplify and enhance computational efficiency, Kipf et al. approximated the l+1 layer network in ChebyNet using a first‐order approach [38]:

(7) $$H^{l+1}=\sigma \left({\hat{D}}^{-\frac{1}{2}}\hat{A}{\hat{D}}^{-\frac{1}{2}}H^{\left(l\right)}W^{\left(l\right)}\right),$$

H pertains to the properties of nodes within the graph, while W signifies the learnable network parameter. $\hat{A}=A+I_{n},{\hat{D}}_{\mathrm{ii}}=\sum j{\hat{A}}_{\mathrm{ij}}$, and the $\sigma \left(\cdot \right)$ is a non‐linear activation function.

Our proposed GCN model utilizes two‐layer convolutions for each pattern graph, with the Rectified Linear Unit (ReLU) serving as the activation function in each convolutional layer. The overall forward propagation is defined by the following formula:

(8) $$f\left(X,A\right)=\text{ReLU}\left[\hat{A}\text{ReLU}\left(\hat{A}XW^{\left(0\right)}\right)W^{\left(1\right)}\right],$$

where $f\left(X,A\right)\in R^{n\times d},$ with $X\in \left\{X_{\mathrm{PC}},X_{\mathrm{SR}},X_{\mathrm{GCM}}\right\}$ and $A\in \left\{A_{\mathrm{PC}},A_{\mathrm{SR}},A_{\mathrm{GCM}}\right\},$ d denotes the dimension of the output features. The weight matrix $W^{\left(0\right)}$ represents the learnable weights of the first convolutional layer, while $W^{\left(1\right)}$ represents the weight matrix of the second convolutional layer.

We employ multiple GCN models to predict category labels for entire graphs, leveraging both the intrinsic structure of the graphs and the detailed information contained within their nodes. The convolutional layers are critical in this framework, as they enable the formation of progressively complex and refined graph representations. These layers systematically capture and process the intricate connection patterns and node‐specific features, enhancing the model's capacity to discern subtle relationships and dependencies within the graph. In tasks involving graph classification, it is common to use a readout layer to generate a representation at the level of the entire graph. Inspired by Lee et al. [41], we employ both maximum and average pooling operations to aggregate node features, yielding fixed‐size vectors for the three patterns derived from a single subject. The readout layer is then formulated as follows:

(9) $$F=\frac{1}{n}\sum_{i=1}^{N}f_{i}\left(X,A\right)\|{\max_{i=1}}^{N}f_{i}\left(X,A\right),$$

here, $f_{i}\left(X,A\right)$ denotes the i− th ROI feature vector obtained through convolution, and ‖ represents the concatenation that consolidates these feature vectors from different ROIs into one unified vector. Consequently, we obtain 64‐dimensional vectors representing graphs with various patterns: $F_{k}={\left[{F_{1k}}^{\left(64\right)},{F_{2k}}^{\left(64\right)},\ldots ,{F_{\mathrm{Nk}}}^{\left(64\right)}\right]}^{T}$, where k=1,2,3. These representation vectors, denoted as $F_{m1},\;F_{m2},\text{and}\;F_{m3}$, correspond to the graph representations of different patterns for the m− th subject, where m ranges from 1 to N.

#### 3.3.3 Classification and Integration of Features From Various Patterns

We aggregate the graph‐level representation vectors obtained from the preceding readout operation by treating each vector with equal importance. This process results in the formation of a novel 192‐dimensional graph representation vector for each subject, given by $\left(F_{1}\|F_{2}={\left[F_{1}^{\left(192\right)},F_{2}^{\left(192\right)},\ldots ,F_{N}^{\left(192\right)}\right]}^{T}\right)$. By combining these vectors, we ensure that the final representation encapsulates a comprehensive and balanced summary of the graph's features, which is essential for subsequent analyses and applications. This vector is then processed through three fully connected layers to further explore the feature information within the graph. Ultimately, the final classification is performed based on the output from the final fully connected layer.

### 3.4 Implementation Details

The MFCP model was implemented in PyTorch and trained on an NVIDIA GeForce RTX 4090 GPU (24 GB VRAM). The architecture comprises three parallel GCN modules, each with two 32‐neuron graph convolutional layers and a readout layer, followed by three fully connected layers (192, 32, and 16 neurons). ReLU activation was applied to all layers. Dropout (rate = 0.5) was used to prevent overfitting. We optimized the model using the Adam optimizer with a learning rate of 0.0025 and weight decay of 0.00001. The detailed information is given in Table 2.

**TABLE 2:** Computing environment and software configuration.

Item	Specification
Operating system	Ubuntu 22.04
CPU	Intel i9‐13900K
RAM	64 GB
GPU	NVIDIA GeForce RTX 4090
CUDA version	12.4
Python version	3.9.19
PyTorch version	2.4.1

## 4 Experiments and Results

### 4.1 Experimental Settings

For the primary within‐site evaluation on Site 20, we implemented a randomized and stratified partitioning strategy, allocating 70% of the data for training, 10% for validation, and the remaining 20% for testing to preserve the class distribution between the MDD and NC groups in each subset. After each training epoch, validation was conducted to identify and save the model parameters that achieved optimal performance. The selected model was then evaluated on the independent test set, and the final performance metrics were reported accordingly. All hyperparameters were selected exclusively based on the training and validation sets, and the test set was used only for final evaluation. To ensure fair comparison, identical data partitioning and training protocols were applied when benchmarking the proposed MFCP against alternative methodologies. For Site 20, this procedure was repeated 30 times with different random seeds, and the mean performance across these repeated experiments was reported. Additionally, a 5‐fold cross‐validation was performed on Site 20 to further ensure the robustness of model training and hyperparameter selection. To evaluate cross‐site generalizability, the model trained and optimized on Site 20 was subsequently applied to the independent Site 21 dataset for external validation.

To gauge the efficacy of various approaches, we adopted five performance metrics: Accuracy, Recall, Precision, F1‐score, and the Area Under the ROC Curve (AUC). These metrics offer a comprehensive evaluation of the model's efficacy. The metrics are defined as follows: True Positives (TP) denote correct identifications of positive instances, True Negatives (TN) are accurate identifications of negative instances, False Positives (FP) are misidentified positive instances, and False Negatives (FN) are misidentified negative instances. The first four metrics are calculated based on these definitions as follows:

(10) $$\text{Accuracy}=\frac{\mathrm{TP}+\mathrm{TN}}{\mathrm{TP}+\mathrm{FN}+\mathrm{FP}+\mathrm{TN}},$$

(11) $$\text{Precision}=\frac{\mathrm{TP}}{\mathrm{TP}+\mathrm{FP}},$$

(12) $$\text{Recall}=\frac{\mathrm{TP}}{\mathrm{TP}+\mathrm{FN}},$$

(13) $$F1-\text{Score}=\frac{2*\text{Precision}*\text{Recall}}{\text{Precision}+\text{Recall}},$$

the model's classification performance improves as the metrics increase. Additionally, the ROC curve is plotted with the True Positive Rate (TPR) on the y‐axis and the False Positive Rate (FPR) on the x‐axis. The AUC measures the probability that the classifier will rank positive cases higher than negative ones. A higher AUC value, approaching 1, signifies a better ability of the model to distinguish between cases.

**FIGURE 2:**
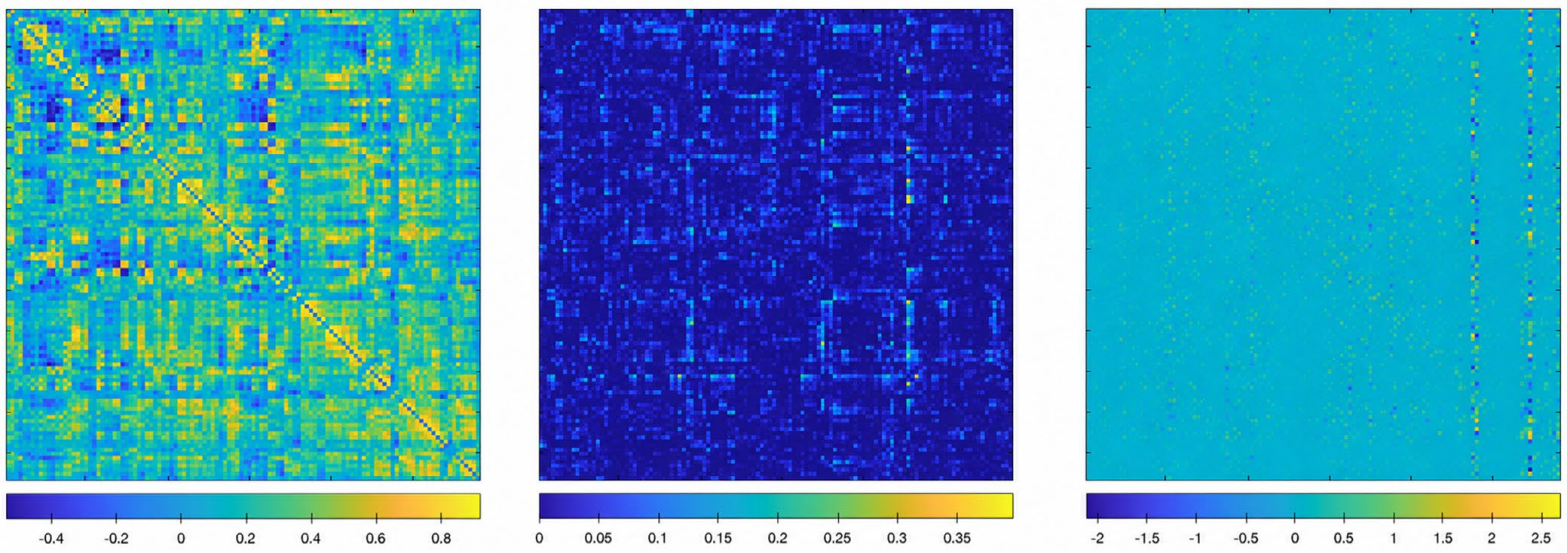
The connection networks derived from the SR, PC, and GCM methods.

Figure 2 illustrates FCN adjacency matrices derived from SR, PC, and GCM techniques. This figure highlights the different brain connection topologies captured by various estimation methods, reflecting the distinct statistical information or relationships among ROIs that each method identifies. Additionally, when constructing FCNs with different threshold settings, the resulting networks also display significant variations. This highlights the importance of carefully considering the choice of estimation method and threshold setting when constructing FCNs for a given dataset. The diverse range of connection weights and network structures produced by these variations underscores the need for thorough analysis and consideration to accurately capture the underlying relationships. In the sensitivity analysis below, we also analyze the influence of different thresholds on the performance of the model.

### 4.2 Methods for Comparison

#### 4.2.1 Ablation Study on Site 20

We juxtaposed our MFCP approach against a spectrum of configurations, ranging from individual to combined partial connection patterns, encompassing: (1) PC, (2) SR, (3) GCM, (4) PC with SR, (5) PC with GCM, and (6) SR with GCM.

Each single‐pattern brain network model consists of one GCN module and three fully connected layers. The GCN module is comprised of a pair of convolutional layers capped with a readout layer [42]. Nonlinear activation functions are utilized in both the convolutional and fully connected layers. Each graph convolutional layer contains 32 neurons. Within the three fully connected layers, there are 64, 32, and 16 neurons, respectively. Classification is carried out by the final layer, which contains two neurons.

The parameter configurations for the three partially connected brain networks align with those used for single‐mode connected networks. However, this approach requires an additional GCN module to handle the extra input. Consequently, the first fully connected layer is expanded to 128 neurons, compared to the single‐mode network.

To ensure a uniform assessment, all seven comparative methods uniformly apply a dropout rate of 0.5 across their fully connected layers. The training process employs the Adam optimizer with a cross‐entropy loss function, using a learning rate of 0.0025 and a regularization factor of 0.00001, extended over 150 epochs. The specific parameters are shown in Table 3.

**TABLE 3:** Network configurations and training settings under different pattern settings.

Setting	Single pattern	Two patterns	Three patterns (MFCP)
Number of GCN networks	1	2	3
Fully connected layers	[64, 32, 16, 2]	[128, 32, 16, 2]	[192, 32, 16, 2]
Dropout	0.5		
Optimizer	Adam		
Learning rate	0.0025		
Weight decay	1 × 10^−5^		
Epochs	150		

#### 4.2.2 Cross‐Site Validation

To comprehensively evaluate the generalizability of the proposed MFCP framework across different sites, we further conducted cross‐site validation experiments where models were trained on Site 20 and directly tested on the independent Site 21 dataset. For this cross‐site scenario, we benchmarked MFCP against several state‐of‐the‐art domain adaptation methods and baseline approaches, including BC‐SVM [43], LE‐SVM [44], MNSFS [20], GIN [45], ST‐GCN [46], CORAL [47], DANN [48], and AUFA [49]. These comparative methods were selected to represent diverse strategies for addressing domain shift in neuroimaging data, namely BC‐SVM, LE‐SVM, and MNSFS as traditional feature‐based classifiers, alongside GIN, ST‐GCN, CORAL, DANN, and AUFA as advanced deep learning and domain adaptation techniques. For cross‐site evaluation, MFCP was trained exclusively on Site 20 and tested on Site 21 without using Site 21 labels during training. The results of the competing methods were taken from previously published studies for reference.

### 4.3 Classification Results

**FIGURE 3:**
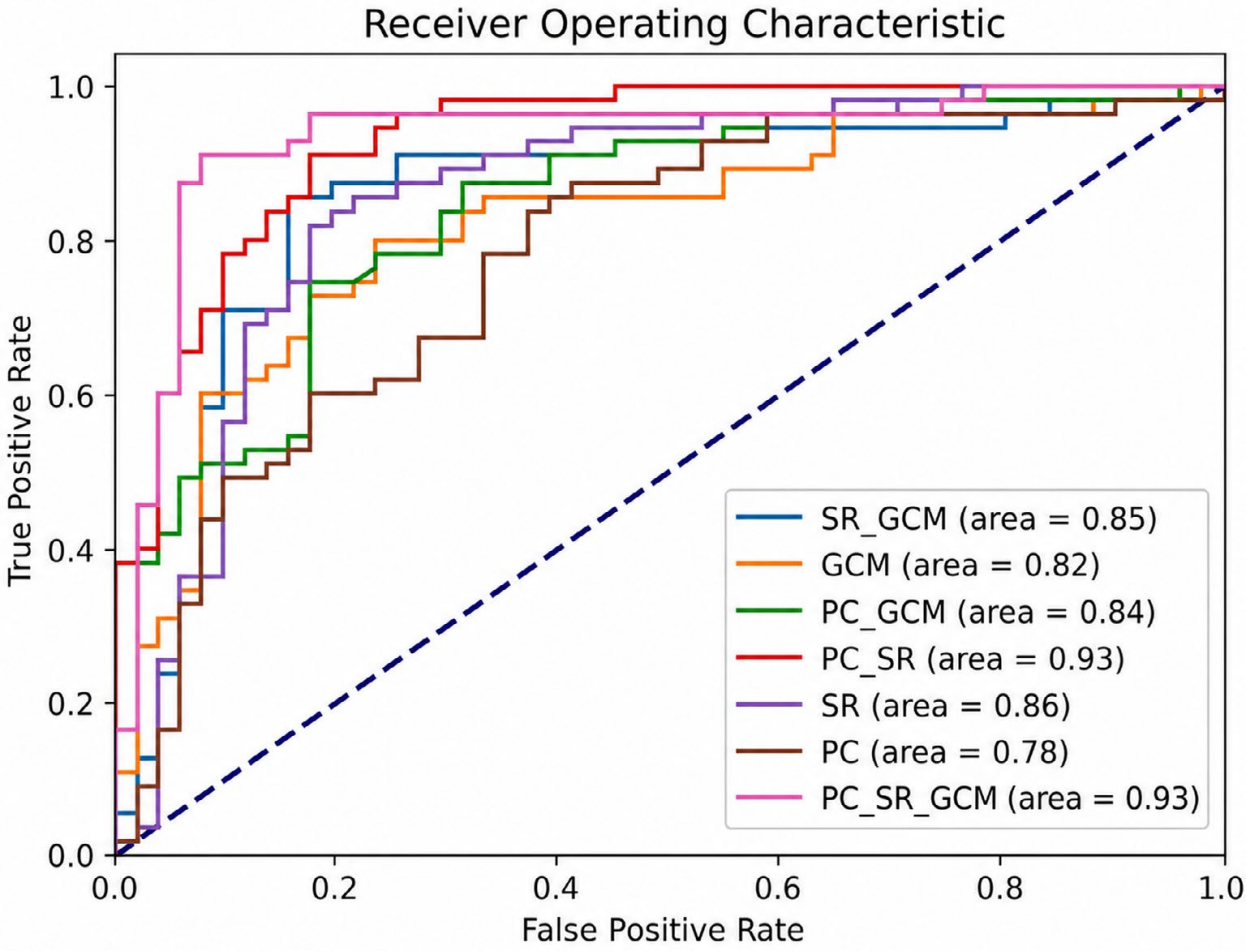
ROC curves of seven methods.

The results of classifying MDD and NC using our MFCP method and six other techniques are presented in Table 4, with performance metrics reported as mean ± standard deviation over 30 independent repetitions to ensure statistical reliability. Figure 3 illustrates the ROC curves for these methods. From these figures, we can draw several important conclusions. Importantly, utilizing dual connection patterns performs significantly better than using a single connection pattern, highlighting the benefit of integrating different types of connection patterns. Additionally, performance improves with the fusion of more connection patterns. The classifier integrating three unique connection patterns outperforms others, underscoring the prowess of MFCP.

**TABLE 4:** Comparative performance of various models in the classification of MDD.

	PC	GCM	SR	PC_SR	PC_GCM	SR_GCM	PC_SR_GCM
Accuracy	0.7264 ± 0.0198	0.7547 ± 0.0148	0.8113 ± 0.0283	0.8302 ± 0.0353	0.7736 ± 0.0199	0.8302 ± 0.0290	**0.8774 ± 0.0282**
Precision	0.6912 ± 0.0508	0.7736 ± 0.0185	0.7869 ± 0.0345	0.8936 ± 0.0334	0.7541 ± 0.0346	0.8491 ± 0.0378	**0.8621 ± 0.0206**
Recall	0.8545 ± 0.0301	0.7455 ± 0.0156	0.8727 ± 0.0211	0.7636 ± 0.0446	0.8364 ± 0.0182	0.8182 ± 0.0328	**0.9091 ± 0.0378**
F1‐score	0.7642 ± 0.0155	0.7593 ± 0.0137	0.8276 ± 0.0261	0.8235 ± 0.0338	0.7931 ± 0.0177	0.8333 ± 0.0262	**0.885 ± 0.0273**
AUC	0.7793 ± 0.0084	0.8185 ± 0.0238	0.856 ± 0.0302	0.9308 ± 0.0190	0.8433 ± 0.0170	0.8542 ± 0.0110	**0.9326 ± 0.0296**

To further validate the robustness of the proposed framework, we additionally performed 5‐fold cross‐validation on Site 20. The detailed performance metrics for each fold and the averaged results are summarized in Table 5, demonstrating consistent performance across different data partitions.

**TABLE 5:** Performance of MFCP via 5‐fold cross‐validation on Site 20.

Metric	Mean ± Std	Fold 1	Fold 2	Fold 3	Fold 4	Fold 5
Accuracy	0.8630 ± 0.0155	0.8505	0.8785	0.8785	0.8679	0.8396
Precision	0.8851 ± 0.0324	0.8727	0.8929	0.9388	0.8387	0.8824
Recall	0.8546 ± 0.0444	0.8421	0.8772	0.8214	0.9286	0.8036
F1‐score	0.8682 ± 0.0166	0.8571	0.8850	0.8762	0.8814	0.8814
AUC	0.9330 ± 0.0123	0.9179	0.9481	0.9471	0.9246	0.9275

Furthermore, to assess the generalizability of MFCP across independent data collection sites, we performed cross‐site validation by training the proposed framework on Site 20 and directly evaluating it on the independent Site 21 dataset. As presented in Table 6, the results of the competing methods were taken from previously published studies. Accordingly, differences in preprocessing pipelines may affect the direct comparability of these results. Despite this limitation, MFCP still demonstrates favorable performance in the cross‐site setting. Moreover, the Mean and Ensemble results in Table 6 were obtained by applying the five models trained in the 5‐fold cross‐validation on Site 20 to Site 21. Specifically, Mean refers to the average performance of the five fold‐specific models on Site 21, whereas Ensemble refers to the performance of their ensemble prediction. These findings further support the robustness and cross‐site generalization ability of the proposed method.

**TABLE 6:** Cross‐site generalization performance comparison (Site 20 → Site 21).

Method	Accuracy	Precision	Recall	F1‐score	AUC
BC‐SVM	0.51	0.55	0.63	0.59	0.49
LE‐SVM	0.52	0.56	0.64	0.59	0.52
MNSFS	0.48	0.56	0.29	0.38	0.49
GIN	0.52 ± 0.04	0.57 ± 0.03	0.55 ± 0.13	0.55 ± 0.08	0.51 ± 0.03
ST‐GCN	0.52 ± 0.05	0.56 ± 0.04	0.60 ± 0.18	0.57 ± 0.08	0.49 ± 0.04
CORAL	0.54 ± 0.02	0.59 ± 0.01	0.58 ± 0.02	0.59 ± 0.01	0.56 ± 0.03
DANN	0.56 ± 0.03	0.62 ± 0.06	0.56 ± 0.13	0.57 ± 0.04	0.58 ± 0.06
AUFA	0.63 ± 0.03	0.66 ± 0.03	0.73 ± 0.06	0.69 ± 0.03	0.62 ± 0.04
Mean	0.64 ± 0.01	0.67 ± 0.01	0.66 ± 0.03	0.67 ± 0.01	0.68 ± 0.01
Ensemble	0.64	0.68	0.66	0.67	0.68

### 4.4 Visualization of Network Features

**FIGURE 4:**
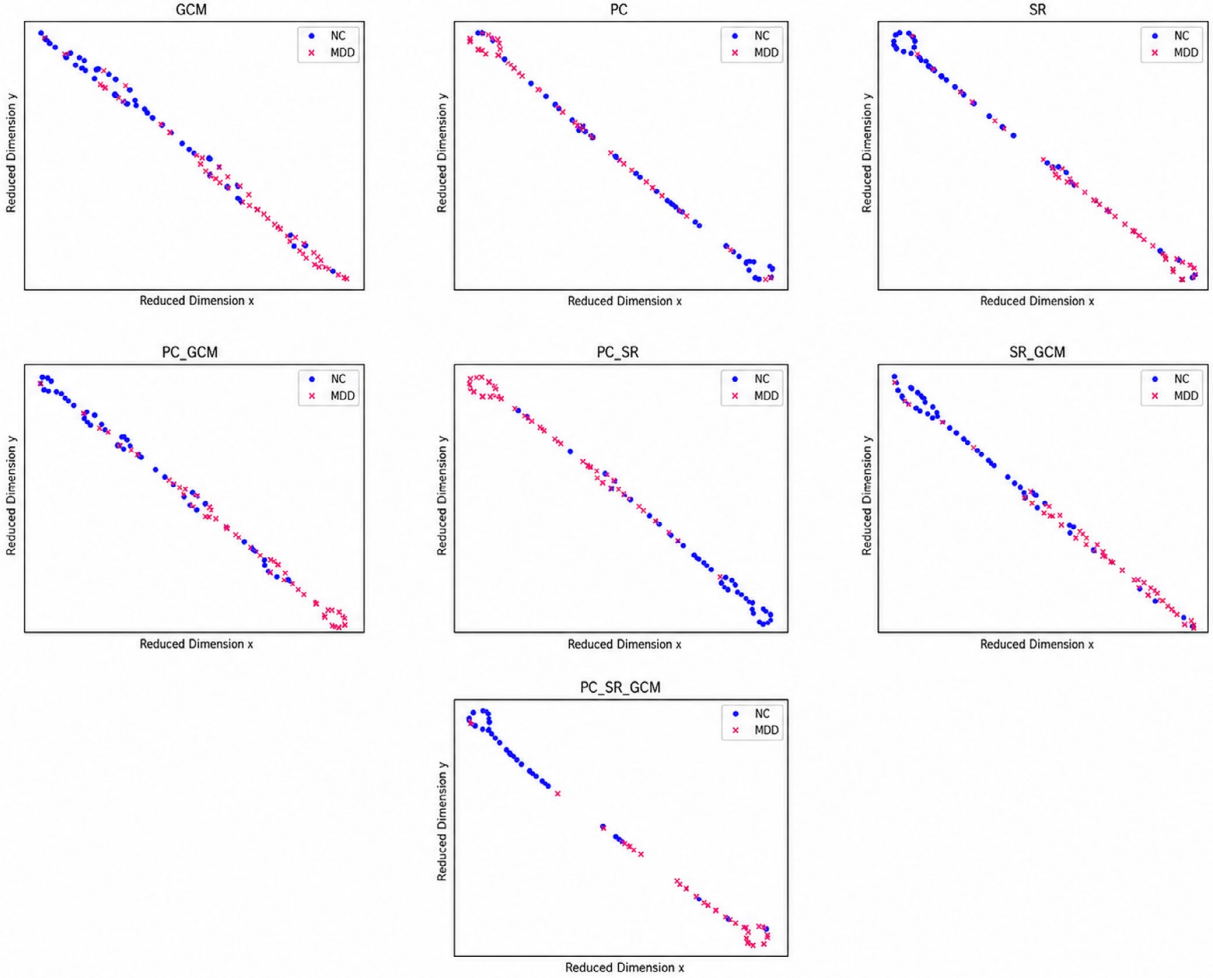
t‐SNE visualization of features derived from seven connectivity modalities.

We employed the t‐Distributed Stochastic Neighbor Embedding (t‐SNE) algorithm [50] to reduce the dimensionality of FCNs’ features obtained from seven distinct connection groups into a two‐dimensional space. This dimensionality reduction technique allowed us to investigate the distribution of FCN characteristics derived from various methods more effectively. Figure 4 illustrates the within‐site clustering performance on Site 20, revealing that the integration of additional connection modalities markedly intensifies the separation between MDD and NC groups. When two modalities are utilized, the separation between different sample types becomes considerably more pronounced, while samples of the same type are clustered more closely together. This enhanced separation effect is even more evident when three modalities are employed, suggesting that the incorporation of diverse connection patterns provides a more detailed and discriminative representation of the data.

**FIGURE 5:**
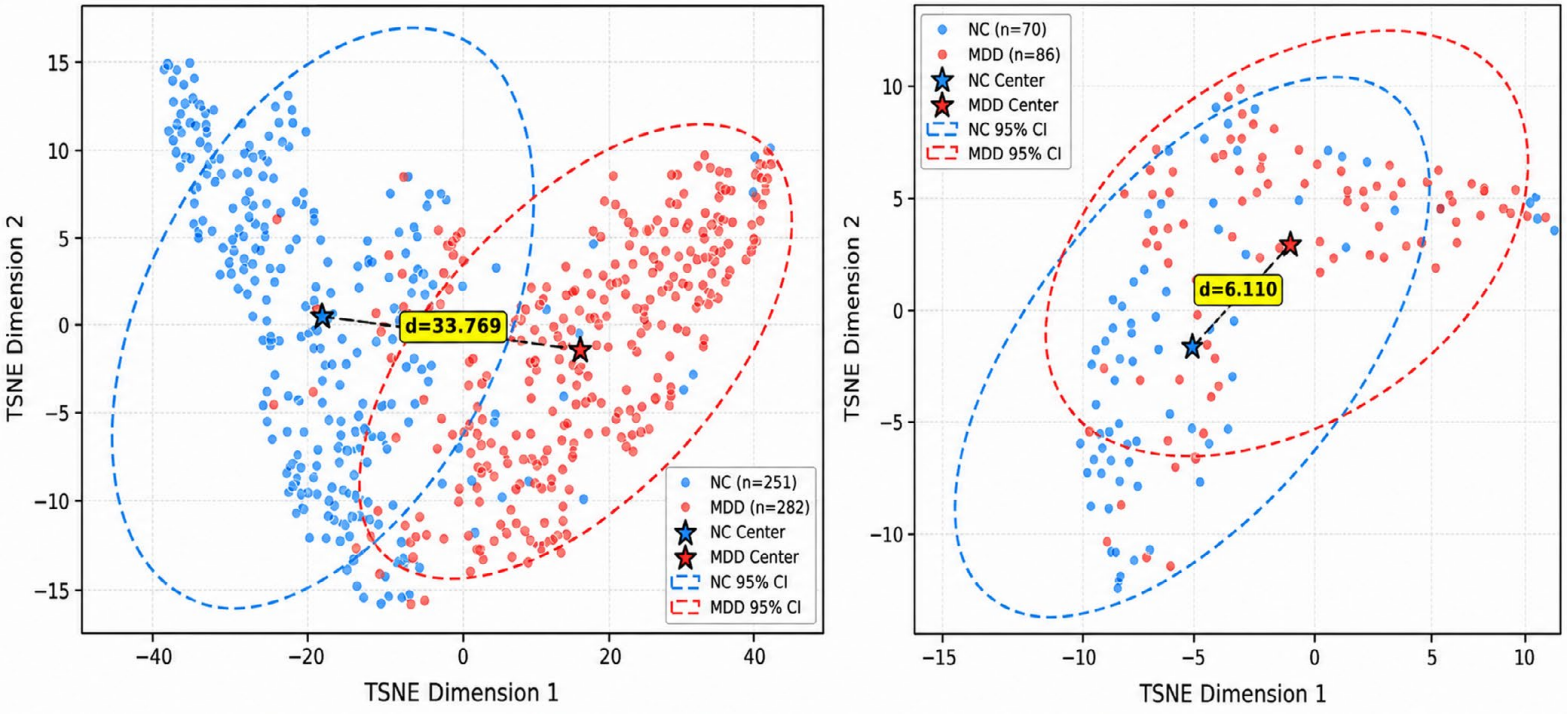
Cross‐site t‐SNE visualization of domain‐invariant features extracted by MFCP.

To further investigate the cross‐site generalizability of the learned representations, Figure 5 presents the t‐SNE visualization of features extracted from Site 20 and Site 21, respectively. The left of Figure 5 illustrates the feature distribution on Site 20, where the NC and MDD groups exhibit clear separation with a center distance of 33.769, indicating distinct clustering patterns for each diagnostic category. The right of Figure 5 demonstrates the feature distribution on Site 21, the independent test set, where the two groups maintain separation with a center distance of 6.110. Although the two groups are less well separated on Site 21 than on Site 20, the result is still encouraging.

**FIGURE 6:**
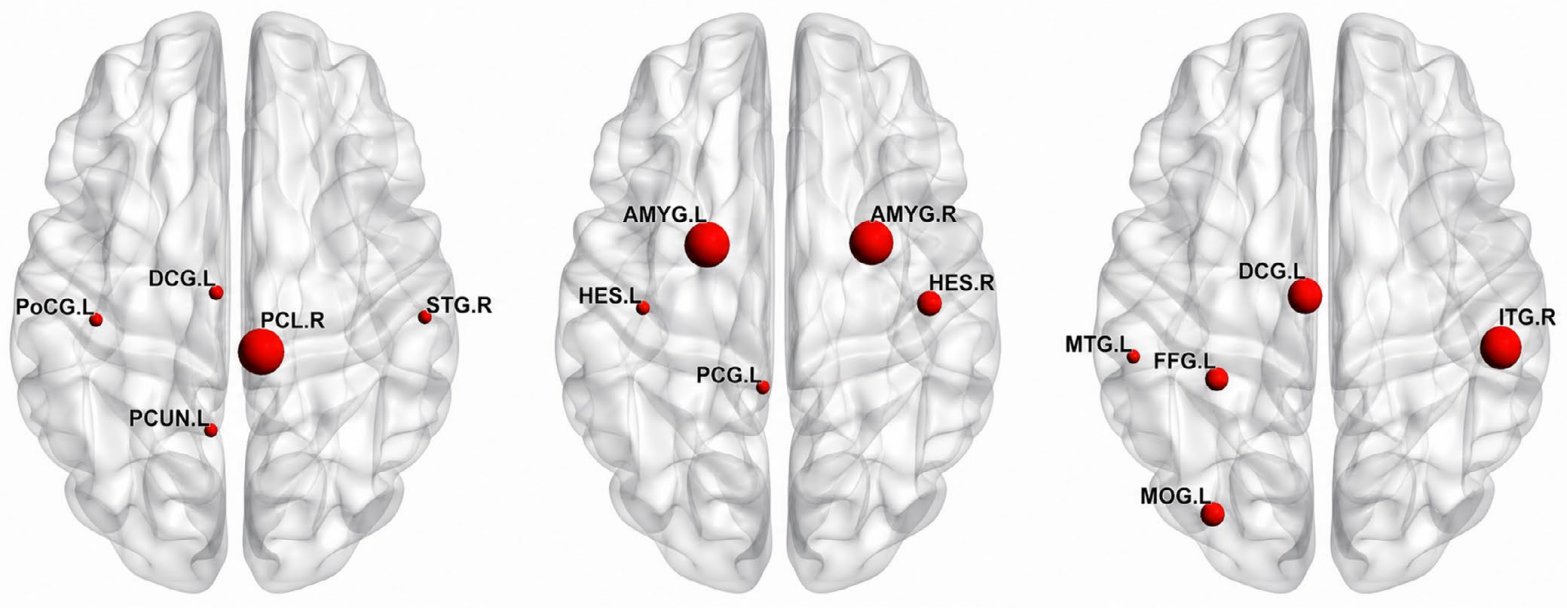
Most discriminative brain regions identified from PC, SR, and GCM (from left to right).

Additionally, we used Grad‐CAM to obtain the most discriminative node for MDD, as shown in Figure 6, to elucidate the connections between the extracted features and different brain regions across three distinct connection patterns. Specifically, we compute node‐level importance using Grad‐CAM on the last graph convolution layer of each pattern‐specific branch, prior to the readout pooling operation. Let $H\in {\mathbb{R}}^{N\times F}$ denote the node feature map produced by this layer, where N=116,which corresponds to the number of ROIs and F=32 is the feature dimension. For a given target class c, we take gradients of the pre‐softmax prediction logit $y^{c}$ with respect to H, i.e., $\partial y^{c}/\partial H$, to obtain class‐discriminative gradients. Following the Grad‐CAM formulation, channel‐wise importance weights are computed by averaging the gradients over the node dimension:

(14) $$\begin{array}{c} \alpha_{k}^{c}=\frac{1}{N}\sum_{i=1}^{N}\frac{\partial y^{c}}{\partial H_{i,k}} \end{array},$$

where $\alpha_{k}^{c}$ represents the importance of feature channel k for class c. The node‐level importance score for ROI i is then calculated as:

(15) $$\begin{array}{c} S_{i}^{c}=\text{ReLU}\left(\sum_{k=1}^{F}\alpha_{k}^{c}H_{i,k}\right), \end{array}$$

which ensures that only positively contributing features are retained. The resulting scores are normalized within each subject to facilitate comparison across ROIs.Since each graph node directly corresponds to an anatomical ROI, the node‐level Grad‐CAM scores are directly interpreted as ROI‐level importance for each subject. In this visualization, the size of each sphere represents the significance of a feature's contribution. For each pattern, the top five ROIs with the highest importance scores across multiple runs were selected for visualization. Figure 6 highlights intriguing variations in the contributions of brain regions associated with each pairwise estimation derived from the FCN methods. These variations underscore the distinct connection patterns captured by our approach, reflecting how different brain regions interact under various conditions. As shown in Figure 6, the discriminative nodes vary substantially across different connectivity patterns, which further confirms the necessity of fusing information from different connection patterns.

In addition, as shown in Table 7, several brain regions highlighted by Grad‐CAM across different connectivity patterns are consistent with prior neuroimaging findings in MDD, particularly regions associated with the default mode network and limbic‐related systems. These regions have been widely reported to play key roles in emotional regulation, self‐referential processing, and cognitive control, which are commonly disrupted in MDD. This consistency with existing literature provides neurobiological support for the relevance of the features identified by the proposed model.

**TABLE 7:** High‐contribution ROIs identified by Grad‐CAM.

ROI Abbreviation	Full Name	Primary Functional Network
PoCG.L	Postcentral gyrus	Sensorimotor Network (SMN)
DCG.L	Median cingulate and paracingulate gyri	Salience Network (SN)
PCL.R	Paracentral lobule	Sensorimotor Network (SMN)
PCUN.L	Precuneus	Default Mode Network (DMN)
STG.R	Superior temporal gyrus	Auditory Association Network
AMYG.L	Amygdala	Limbic Network
AMYG.R	Amygdala	Limbic Network
HES.L	Heschl gyrus	Primary Auditory Network
HES.R	Heschl gyrus	Primary Auditory Network
PCG.L	Posterior cingulate gyrus	Default Mode Network (DMN)
MTG.L	Middle temporal gyrus	Default Mode/Ventral Attention Network
FFG.L	Fusiform gyrus	Visual Network (Ventral Stream)
ITG.R	Inferior temporal gyrus	Visual Network (Ventral Stream)
MOG.L	Middle occipital gyrus	Visual Network
DCG.L	Median cingulate and paracingulate gyri	Salience Network (SN)

The observed differences in highlighted regions across connection patterns can be attributed to the distinct characteristics of each pattern. Pearson correlation primarily captures synchronous co‐fluctuations between regions, sparse representation emphasizes conditional and discriminative dependencies, whereas Granger causality mapping characterizes directed effective interactions. As a result, each pattern tends to emphasize different but complementary aspects of brain connectivity, leading to distinct sets of discriminative regions identified by Grad‐CAM. When multiple connection patterns are jointly used as input, the proposed model learns a fused representation that integrates information across patterns rather than providing explicit pattern‐wise attribution.

### 4.5 Sensitivity Analysis

**FIGURE 7:**
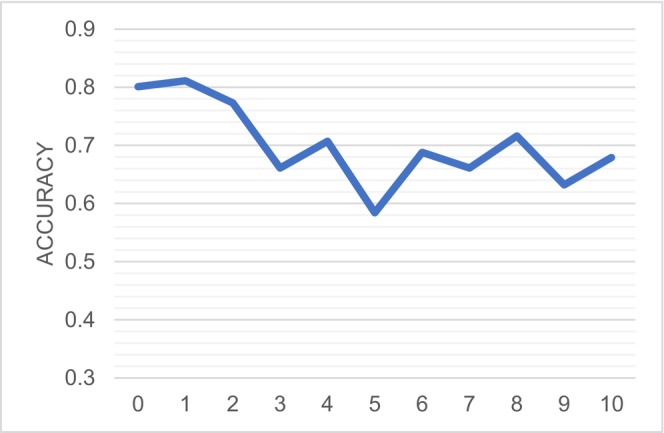
Sensitivity analysis of different λ for SR.

The performance of the Sparse Representation (SR) in the final stage is highly influenced by the hyperparameter λ. To understand its impact, we conducted a sensitivity analysis on the classification results using different values of λ in our experiments. As depicted in Figure 7, the highest accuracy of 81.1% was achieved when λ was set to 1. This finding indicates that the selection of λ is critical for optimizing model performance. Consequently, based on these results, we chose λ = 1 for constructing the FCN of patients, which served as the model input in our implementation. This sensitivity analysis highlights the importance of tuning hyperparameters to enhance the accuracy and robustness of the model. By identifying the optimal value of λ, we ensured that our model effectively captures the relevant features and provides reliable classification results.

**FIGURE 8:**
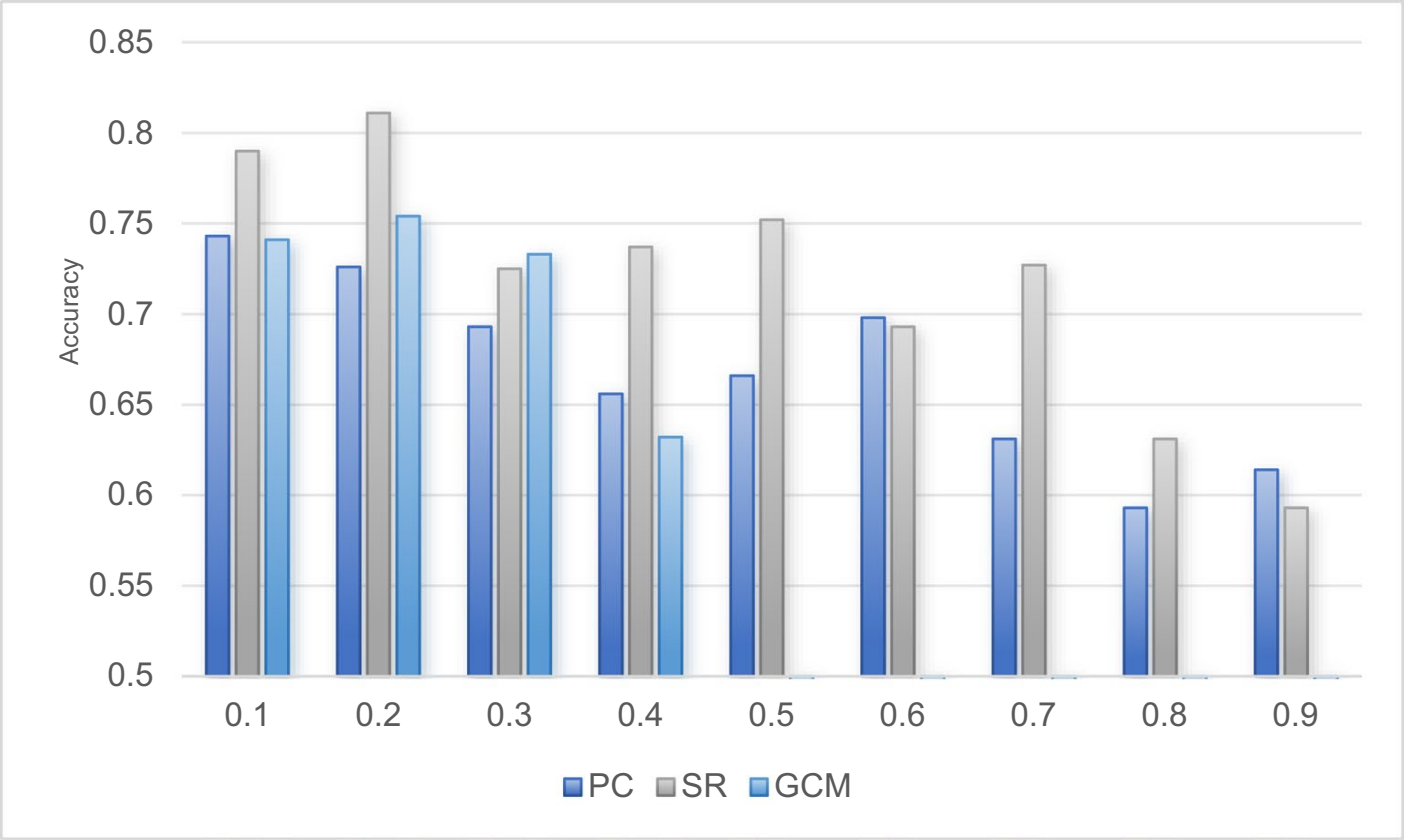
Sensitivity analysis of different thresholds for single‐pattern models.

We also investigated the correlation between performance and the estimated FCN inputs using different thresholds in a single connection mode. As shown in Figure 8, the optimal performance for Pearson's Correlation (PC), Sparse Representation (SR), and Granger Causality Mapping (GCM) was achieved at thresholds of 0.1, 0.2, and 0.2, respectively. These thresholds resulted in accuracies of 74.3%, 81.1%, and 75.4%. Our analysis revealed that for various FCN estimation methods, the threshold yielding the best performance did not always correspond to the lowest value. This suggests the presence of noisy or redundant connections within the brain FCN, which could potentially impede model performance. Identifying the appropriate threshold is thus crucial for minimizing the influence of these extraneous connections and enhancing the overall accuracy of the model. Furthermore, it is noteworthy that the GCM accuracy drops to zero at a threshold of 0.5 or higher. This is due to the impracticality of constructing an FCN matrix beyond this threshold, as excessively high thresholds may eliminate essential connections, rendering the FCN representation ineffective. This investigation underscores the importance of carefully selecting thresholds when estimating FCNs to ensure optimal model performance. By doing so, we can better filter out irrelevant connections and focus on the most informative ones, thereby improving the robustness and reliability of our classification results.

## 5 Discussion

In this work, we introduce the MFCP framework for automated MDD diagnosis using rs‐fMRI. Unlike single‐pattern approaches that capture limited aspects of brain connectivity, MFCP integrates Pearson's Correlation, Sparse Representation, and Granger Causality. This multi‐view strategy yields substantial performance gains, with accuracy increasing from ~73% to 87.74%, demonstrating that complementary connection patterns are essential for capturing the multifaceted nature of MDD pathophysiology.

We conducted rigorous validation to assess stability and generalizability. First, 5‐fold cross‐validation on Site 20 yielded a mean accuracy of 0.8630, consistent with the single‐split evaluation results and indicating stable performance across varying data partitions. Second, cross‐site validation achieved an accuracy of approximately 64%, showing competitive performance relative to domain adaptation methods such as CORAL, DANN, and AUFA. The t‐SNE visualizations suggest that MFCP learns relatively domain‐invariant features, maintaining MDD‐NC separation despite reduced inter‐class distance on Site 21.

Grad‐CAM analysis reveals the neurobiological basis of MFCP's decisions. Distinct patterns emphasized complementary regions: PC highlighted sensorimotor and default mode networks; SR emphasized limbic structures and auditory cortex; GCM revealed visual‐associative and salience regions. These findings align with established MDD pathology, particularly the disrupted interactions among the default mode, limbic, and salience networks.

Despite strong performance, limitations remain. The cross‐site accuracy drop indicates persistent domain shift challenges requiring advanced domain generalization techniques for seamless clinical deployment. Additionally, our simple concatenation fusion strategy may be suboptimal compared to adaptive attention mechanisms.

In summary, we focus on multi‐site validation and biological interpretability to assess MFCP's clinical potential. Future work will integrate domain generalization techniques to enhance real‐world diagnostic utility.

## 6 Conclusion

We propose the MFCP for automated MDD diagnosis, integrating Pearson Correlation, Sparse Representation, and Granger Causality to capture complementary aspects of brain connectivity. Evaluated on 533 subjects from REST‐MDD Site 20, MFCP achieved 87.74% accuracy and 0.9326 AUC, significantly outperforming single‐pattern methods. Furthermore, 5‐fold cross‐validation confirmed model stability, while cross‐site validation demonstrated competitive generalizability in comparison with existing domain adaptation techniques.

Grad‐CAM analysis revealed that MFCP identifies neurobiologically meaningful patterns, emphasizing the default mode network, limbic system, and salience network, regions consistently implicated in MDD pathophysiology. This multi‐pattern approach provides a trans‐network characterization that single methods cannot achieve, offering both high classification performance and clinical interpretability.

While cross‐site performance indicates remaining domain shift challenges, MFCP provides a promising framework for rs‐fMRI‐based MDD identification. Future work will integrate advanced domain generalization techniques to enhance clinical deployment.

## Funding

This work was supported by the National Natural Science Foundation of China (62306051), the Taishan Scholars Foundation of Shandong Province (tsqn20250722), the Science and Technology Research Program of Chongqing Municipal Education Commission (KJQN202300718, KJZD‐K202400703), the 2024‐2025 Research Project under the Open Competition Mechanism, School of Medicine, Shanghai University (SHU‐UM‐JBGS‐2025‐1), the Scientific Research Subjects of Shanghai Universal Medical Imaging Technology (UV2024M03, UV2025M05, UV2026M03), and the Natural Science Foundation of Chongqing Municipality (CSTB2023NSCQ‐LZX0092, CSTB2025NSCQ‐GPX0857).

## Disclosure

All authors report no biomedical financial interests or potential conflicts of interest.

## Ethics Statement

All data is acquired from the publicly available dataset.

## Conflicts of Interest

The authors declare no conflicts of interest.

## Data Availability

The data that support the findings of this study are available on request from the corresponding author. The data are not publicly available due to privacy or ethical restrictions.
